# TNFα and IFNγ but Not Perforin Are Critical for CD8 T Cell-Mediated Protection against Pulmonary *Yersinia pestis* Infection

**DOI:** 10.1371/journal.ppat.1004142

**Published:** 2014-05-22

**Authors:** Frank M. Szaba, Lawrence W. Kummer, Debra K. Duso, Ekaterina P. Koroleva, Alexei V. Tumanov, Andrea M. Cooper, James B. Bliska, Stephen T. Smiley, Jr-Shiuan Lin

**Affiliations:** 1 Trudeau Institute, Saranac Lake, New York, United States of America; 2 Center for Infectious Diseases and Department of Molecular Genetics and Microbiology, Stony Brook University, Stony Brook, New York, United States of America; Yale University School of Medicine, United States of America

## Abstract

Septic pneumonias resulting from bacterial infections of the lung are a leading cause of human death worldwide. Little is known about the capacity of CD8 T cell-mediated immunity to combat these infections and the types of effector functions that may be most effective. Pneumonic plague is an acutely lethal septic pneumonia caused by the Gram-negative bacterium *Yersinia pestis*. We recently identified a dominant and protective *Y. pestis* antigen, YopE_69–77_, recognized by CD8 T cells in C57BL/6 mice. Here, we use gene-deficient mice, Ab-mediated depletion, cell transfers, and bone marrow chimeric mice to investigate the effector functions of YopE_69–77_-specific CD8 T cells and their relative contributions during pulmonary *Y. pestis* infection. We demonstrate that YopE_69–77_-specific CD8 T cells exhibit perforin-dependent cytotoxicity *in vivo*; however, perforin is dispensable for YopE_69–77_-mediated protection. In contrast, YopE_69–77_-mediated protection is severely impaired when production of TNFα and IFNγ by CD8 T cells is simultaneously ablated. Interestingly, TNFα is absolutely required at the time of challenge infection and can be provided by either T cells or non-T cells, whereas IFNγ provided by T cells prior to challenge appears to facilitate the differentiation of optimally protective CD8 T cells. We conclude that cytokine production, not cytotoxicity, is essential for CD8 T cell-mediated control of pulmonary *Y. pestis* infection and we suggest that assays detecting Ag-specific TNFα production in addition to antibody titers may be useful correlates of vaccine efficacy against plague and other acutely lethal septic bacterial pneumonias.

## Author Summary

Bacterial pneumonia is one of the most common causes of death worldwide. Pulmonary infection of bacterium *Yersinia pestis*, the causative agent of plague, results in pneumonic plague and is extremely lethal. Mouse models of pulmonary *Y. pestis* infection are considered translational tools for the development of pneumonic plague countermeasures and studies of the basic mechanisms of immune defense against acutely lethal pulmonary bacterial infections. Here, we used several methods to investigate the functions that CD8 T cells exert to confer protection against pulmonary *Y. pestis* infection and evaluated their relative contributions. We found that although CD8 T cells have the ability to kill *Y. pestis*-infected cells, a function called cytotoxicity, this function is not required for CD8 T cells to protect against *Y. pestis* infection. In contrast, protection depends upon the ability of CD8 T cells to produce the cytokines TNFα and IFNγ, and mice whose T cells cannot produce these two cytokines are not protected. Therefore, we conclude that cytokine production, not cytotoxicity, is essential for CD8 T cell-mediated control of pulmonary *Y. pestis* infection and we suggest that assays detecting cytokine production may be useful correlates of vaccine efficacy against plague and other acutely lethal septic bacterial pneumonias.

## Introduction

Plague, one of the world's most deadly infectious diseases, has killed hundreds of millions of humans during three major pandemics [1]. It is caused by the Gram-negative facultative intracellular bacterium *Yersinia pestis*, which is naturally maintained in rodent reservoirs. Fleas transmit *Y. pestis* between rodents and to other mammals. Human infections typically result from fleabites as well, but a pneumonic form of plague can spread from human to human via infectious respiratory droplets. Pneumonic plague is fulminant and nearly always fatal unless treated with antibiotics within 24 h of symptom onset. Although natural outbreaks of pneumonic plague are uncommon, the high mortality rate, small window for treatment, existence of antibiotics-resistant strains, and potential for use as an airborne biological weapon fosters research aimed at the development of effective countermeasures.

Mouse models of pulmonary *Y. pestis* infection are considered translational tools for the development of pneumonic plague countermeasures because the pathology of plague in rodents is highly similar to that observed in humans. Analogous septic pneumonias caused by more common bacteria, including members of the *Klebsiella*, *Streptococcus*, and *Staphylococcus* species, are leading causes of death worldwide [2], [3]. Thus, murine models of plague also provide tools for studying basic mechanisms of immune defense against acutely lethal bacterial infections that seed the human lung and then disseminate to cause septic morbidity.

Ab-based subunit vaccines composed of the *Y. pestis* F1 and LcrV proteins provide rodents and some nonhuman primates with substantial protection against pulmonary *Y. pestis* infection [4]. Despite inducing high titer Ab responses, these vaccines fail to induce adequate protection in all nonhuman primates, most notably in African green monkeys [4], [5], [6]. This observation raises the possibility that Abs may not suffice to protect humans against pneumonic plague. Recent studies indicate T cells also contribute to protection against pulmonary *Y. pestis* infection in mice and the cytokines TNFα, IFNγ and IL-17 are required for optimal T cell-mediated protection [7], [8]. For example, B cell-deficient mice vaccinated with live attenuated *Y. pestis* are protected against lethal challenge, and depleting T cells or neutralizing TNFα and IFNγ at the time of challenge fully abolishes the protection [7]. TNFα and IFNγ also contribute to Ab-mediated protection in wild-type mice: the passive protection conferred by therapeutic administration of F1 and LcrV-specific mAb and the active protection conferred by immunization with a recombinant F1/LcrV vaccine are both abolished by neutralization of TNFα and IFNγ [9], [10]. Together, these findings suggest that pneumonic plague vaccines should also aim to induce cellular immunity that produces cytokines, in addition to inducing Ab-mediated humoral immunity.

CD8 T cells are critical for defense against a variety of pathogens, including viruses, protozoa and bacteria [11], [12]. The effector functions used by CD8 T cells to resist pathogens include secretion of cytokines like TNFα and IFNγ and Ag-specific cytolysis of infected cells [11], [12]. Recently we identified a dominant and protective T cell epitope recognized by *Y. pestis*-specific CD8 T cells [13]. Immunizing wild-type C57BL/6 mice with this single peptide, YopE_69–77_, primes Ag-specific CD8 T cells that suffice to confer protection against lethal pulmonary *Y. pestis* challenge [13]. Moreover, immunizing mice with YopE_69–77_ also elicits a CD8 T cell response that protects against lethal intragastric challenge with *Yersinia pseudotuberculosis*, the enteropathogen from which *Y. pestis* evolved [14]. A prior study showed that CD8 T cells and perforin, a key molecule for cytolysis, are required to protect naïve mice against attenuated *Y. pseudotuberculosis* infection [15]. However, the relative contributions of cytokines and cytolysis to CD8 T cell-mediated anti-*Yersinia* immunity *in vivo* have never been reported. Furthermore, it is not yet clear whether T cells or other cell types produce the TNFα and IFNγ required for effective T cell-mediated defense against plague.

In this study, we examined the effector functions of YopE_69–77_-specific CD8 T cells during pulmonary *Y. pestis* infection, and investigated their relative contributions to protection mediated by YopE_69–77_ immunization. Using a combination of gene-deficient mice, Ab-mediated depletion, cell transfers, and bone marrow chimeric mice, we demonstrate that YopE_69–77_-specific CD8 T cells exhibit perforin-dependent cytotoxicity *in vivo*, but perforin is dispensable for YopE_69–77_-specific CD8 T cell-mediated protection. In contrast, TNFα and IFNγ are critical for protection and YopE_69–77_-mediated protection is severely impaired when production of TNFα and IFNγ by CD8 T cells is simultaneously suppressed.

## Results

### Immunization with YopE_69–77_ confers protection against *Y. pestis*

**Figure 1:**
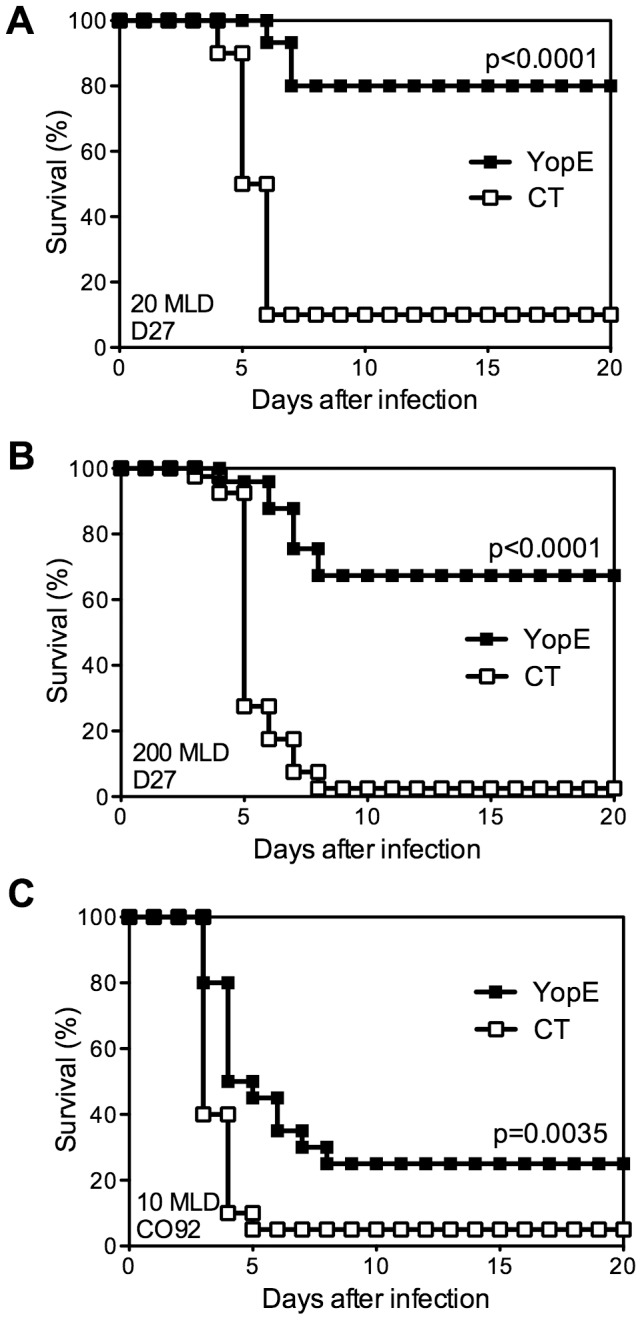
Immunization with YopE_69–77_ peptide protects mice against *Y. pestis*. Wild-type C57BL/6 mice were immunized intranasally with CT adjuvant alone (CT) or CT mixed with YopE_69–77_ peptide (YopE) and then challenged intranasally with (A) 20 MLD (2×10^5^ CFU) or (B) 200 MLD (2×10^6^ CFU) *Y. pestis* strain D27 or (C) 10 MLD (1×10^4^ CFU) *Y. pestis* strain CO92. In comparison with CT–immunized mice (n = 10–40), YopE_69–77_–immunized mice (n = 15–39) exhibited significantly increased survival. Data were pooled from 2–5 independent experiments.

Prior studies established that intranasal immunization with YopE_69–77_, a dominant CD8 T cell epitope of the *Y. pestis* YopE protein, confers C57BL/6 mice with CD8 T cell-mediated protection against pulmonary *Y. pestis* infection [13]; however, the mechanism of protection has yet to be defined. To investigate the relative contributions of cytokines and cytolysis to YopE_69–77_-specific CD8 T cell-mediated protection, we first examined the protective efficacy of YopE_69–77_ immunization. Wild-type C57BL/6 mice were immunized intranasally with adjuvant cholera toxin (CT) alone or adjuvant with 1 µg of YopE_69–77_ and then challenged intranasally with *Y. pestis*. As previously described [13], immunization with YopE_69–77_ conferred protection against 20 median lethal dose (MLD) of *Y. pestis* strain D27, with 80% YopE_69–77_-immunized mice surviving the lethal challenge (Figure 1A). Immunization with YopE_69–77_ also conferred significant protection against a higher dose (200 MLD) challenge of *Y. pestis* strain D27 (Figure 1B), with 67% mice surviving. Moreover, immunization with YopE_69–77_ even sufficed to confer modest yet significant protection against intranasal challenge with fully virulent pigmentation locus-positive *Y. pestis* strain CO92 (Figure 1C). Although 75% of YopE_69–77_-immunized mice ultimately succumbed to the fully virulent challenge, protection was evidenced by both a 1.5-day prolongation in median survival time and an increase in the overall survival rate (Figure 1C). These data indicate that YopE_69–77_ immunization confers significant CD8 T cell-mediated protection against lethal pulmonary challenge with multiple *Y. pestis* strains. To investigate the mechanism of CD8 T cell-mediated defense *in vivo*, we focused on the model employing 20 MLD of *Y. pestis* strain D27 challenge, as this model showed the greatest degree of protection.

### Mice immunized with YopE_69–77_ exhibit perforin-dependent cytotoxic activity

**Figure 2:**
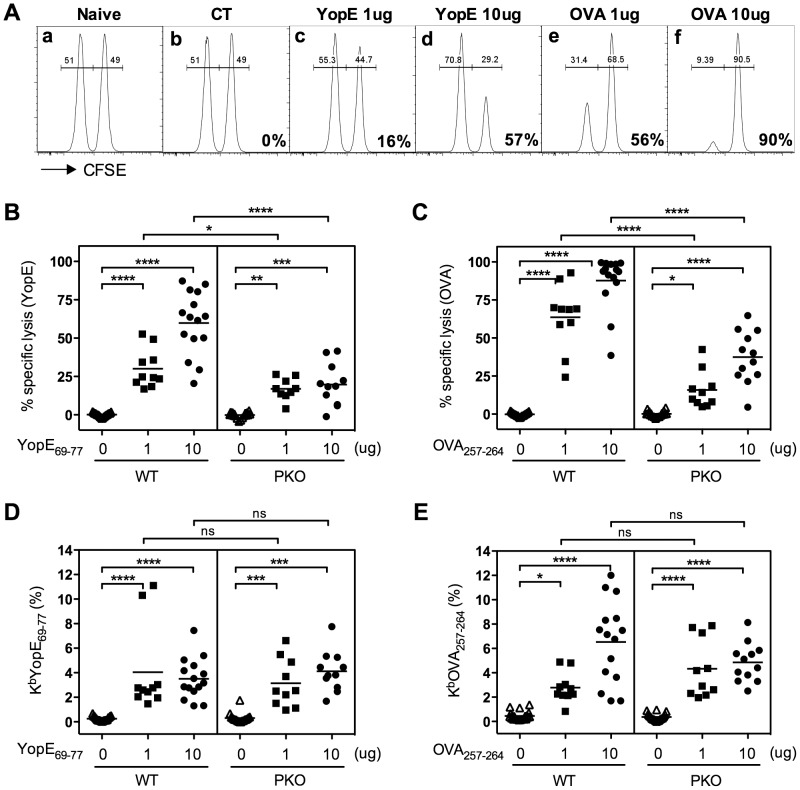
Mice immunized with YopE_69–77_ exhibit perforin-dependent cytotoxic activity. Wild-type (WT) and perforin-deficient (PKO) C57BL/6 mice were immunized with CT adjuvant alone or CT mixed with 1 or 10 µg of YopE_69–77_ or OVA_257–264_ peptides. Splenocytes from naïve congenic WT mice (CD45.1+) that were either pulsed with YopE_69–77_ peptide and labeled with 10 µM of CFSE or pulsed with OVA_257–264_ peptide and labeled with 1 µM of CFSE were mixed together at a 1∶1 ratio and injected into the immunized recipient mice. Splenocytes of recipient mice were then collected 20–22 h later and stained for congenic marker. The target cells (CD45.1+CD45.2−) were gated and the proportion of each CFSE-labeled population was analyzed by flow cytometry. The percent specific lysis was then calculated as described in the Materials and Methods. (A) Representative plots of the flow cytometry analysis of naïve or immunized WT recipient mice. The numbers on the lower right corner of the plots depict the percentage of specific lysis of YopE_69–77_-pulsed target cells or OVA_257–264_-pulsed target cells. (B and C) The percentages of specific lysis of YopE_69–77_-pulsed target cells by YopE_69–77_-immunized mice (B) or OVA_257–264_-pulsed target cells by OVA_257–264_-immunized mice (C). In comparison with WT mice, PKO mice displayed significantly decreased cytotoxicity (one-way ANOVA). Data shown are pooled from 5 independent experiments. (D and E) The percentages of PBL staining positive for CD8 and K^b^YopE_69–77_ (D) or K^b^OVA_257–264_ (E) tetramers two days before the cytotoxicity assay. In comparison with WT mice, PKO mice immunized with the same amount of peptide have comparable Ag-specific CD8 T cell frequencies (one-way ANOVA). Data shown are pooled from 5 independent experiments.

To test whether immunization with YopE elicits a CTL response, we measured the ability of YopE_67–77_-specific CD8 T cells to lyse peptide-pulsed autologous primary lymphocytes using a standard *in vivo* CTL assay [16]. Naïve splenocyte target cells from CD45.1 congenic C57BL/6 mice were pulsed with YopE_69–77_ peptide and labeled with a high concentration of CFSE. Control target cells were pulsed with OVA_257–264_ peptide and labeled with a low concentration of CFSE. The pulsed cells were then mixed at a one-to-one ratio and injected intravenously into wild-type C57BL/6 mice that had been previously immunized. Approximately 20 h later, splenocytes were harvested and the proportion of transferred target cells labeled with high or low levels of CFSE was determined by flow cytometry. The proportion of cells recovered from naïve C57BL/6 mice was used as the comparator (Figure 2A, a).

As expected, near equal proportions of both target cell populations were recovered from mice immunized with CT adjuvant alone, indicating that no specific lysis was taking place (Figure 2A, b). Cells harvested from YopE_69–77_-immunized mice had lower proportions of CFSE^high^ target cells, indicating that the YopE_69–77_-pulsed target cells had been lysed *in vivo* (Figure 2A, c and d). In contrast, cells harvested from OVA_257–264_-immunized mice had lower proportions of CFSE^low^ target cells, indicating that the OVA_257–264_-pulsed target cells were killed (Figure 2A, e and f). Mice immunized with 10 µg of peptide (Figure 2A, d and f) exhibited stronger *in vivo* cytotoxic activity than mice immunized with 1 µg of peptide (Figure 2A, c and e). Together, these data suggest that immunization with YopE_69–77_ primes T cells that have the ability to specifically kill YopE_69–77_-pulsed target cells *in vivo*.

Perforin can play a critical role in CD8 T cell-mediated cytotoxicity, and perforin-deficient mice display increased susceptibility to many viral, bacterial and protozoan infections [11], [12], [17]. To investigate whether perforin contributes to YopE_69–77_-specific CD8 T cell-mediated cytotoxicity, Ag-pulsed target cells were transferred into immunized perforin-deficient mice and the CTL assay was performed. The YopE_69–77_- and OVA_257–264_-immunized perforin-deficient mice showed significantly reduced cytotoxic activity against YopE_69–77_- and OVA_257–264_-pulsed target cells, respectively, in comparison with immunized wild-type mice (Figures 2B and 2C). The reduced cytotoxicity could not be explained by reduced T cell priming, as the peripheral blood of the immunized wild-type and perforin-deficient mice harbored comparable percentages of YopE_69–77_-specific (Figure 2D) or OVA_257–264_-specific (Figure 2E) CD8 T cells. These data demonstrate that YopE_69–77_-specific CD8 T cells have the ability to kill YopE_69–77_-pulsed target cells *in vivo* in a perforin-dependent manner.

### Perforin is dispensable for YopE_69–77_-specific CD8 T cell-mediated protection against *Y. pestis* and *Y. pseudotuberculosis*

**Figure 3:**
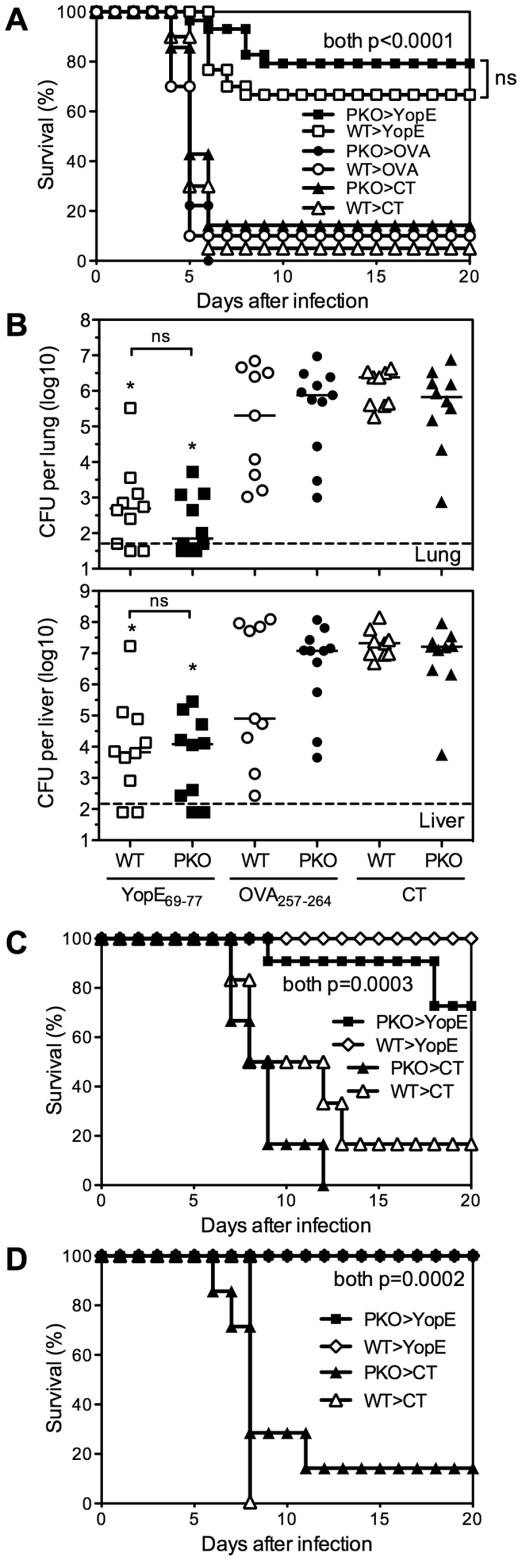
Perforin is dispensable for YopE_69–77_–specific CD8 T cell-mediated protection against *Y. pestis* and *Y. pseudotuberculosis*. Wild-type (WT) and perforin-deficient (PKO) C57BL/6 mice were immunized intranasally with CT adjuvant alone, or CT mixed with YopE_69–77_ or OVA_257–264_ peptides and then challenged with (A and B) 20 MLD (2×10^5^ CFU) *Y. pestis* strain D27 intranasally, (C) 10 MLD (5×10^9^ CFU) *Y. pseudotuberculosis* strain 32777 intragastrically or (D) 10 MLD (1.2×10^2^ CFU) *Y. pseudotuberculosis* strain 32777 intravenously. (A) *Y. pestis* survival data pooled from 3 independent experiments (n = 9–30 mice/group). (B) Day 4 bacterial burden in lung and liver tissues after *Y. pestis* challenge (Kruskal-Wallis test, compared with CT- or OVA_257–264_–immunized PKO or WT mice). Data are pooled from 2 independent experiments (n = 9–11 mice/group). Solid bar depicts median; broken line depicts the limit of detection. (C and D) *Y. pseudotuberculosis* survival data (n = 6–7 mice/group for CT, n = 10–11 mice/group for YopE). Data were pooled from 2 independent experiments.

We next investigated whether perforin is required for protection against lethal *Y. pestis* challenge. Wild-type or perforin-deficient mice were immunized with YopE_69–77_ and then challenged intranasally with 20 MLD of *Y. pestis* strain D27. As shown in Figure 3A, most control mice immunized with adjuvant alone or with control peptide OVA_257–264_ succumbed to *Y. pestis* challenge, whereas most wild-type and perforin-deficient mice immunized with YopE_69–77_ survived. Similar results were observed when mice were immunized with either 1 µg or 10 µg YopE_69–77_ (Figure 3A zand data not shown). Measurements of bacterial burden in lung and liver tissues at day 4 after *Y. pestis* challenge showed significantly decreased numbers of CFU in both the YopE_69–77_-immunized wild-type and perforin-deficient mice, as compared with control mice immunized with adjuvant alone or with OVA_257–264_ (Figure 3B). Neither the survival data nor the bacterial burden data revealed a significant role for perforin in the YopE_69–77_-specific CD8 T cell-mediated protection against *Y. pestis*. Therefore, although the YopE_69–77_-specific CD8 T cells exhibit perforin-dependent cytotoxic activity *in vivo* (Figure 2), perforin is dispensable for protection against lethal *Y. pestis* challenge.

We previously demonstrated that immunization with YopE_69–77_ also protects against lethal intragastric challenge of mice with *Y. pseudotuberculosis* [14]. Other investigators reported that naïve perforin-deficient mice display increased susceptibility to intravenous challenge with an attenuated *Y. pseudotuberculosis* strain [15]. To investigate whether perforin is required for YopE_69–77_-specific CD8 T cell-mediated protection against *Y. pseudotuberculosis*, we immunized wild-type and perforin-deficient mice and challenged intragastrically or intravenously with 10 MLD of virulent *Y. pseudotuberculosis* strain 32777. We observed that both wild-type and perforin-deficient mice immunized with YopE_69–77_ were specifically protected against intragastric and intravenous *Y. pseudotuberculosis* challenge (Figures 3C and 3D, respectively). These data indicate that perforin is also dispensable for YopE_69–77_-specific CD8 T cell-mediated protection against *Y. pseudotuberculosis*.

### TNFα and IFNγ are critical for YopE_69–77_-specific CD8 T cell-mediated protection against *Y. pestis*

**Figure 4:**
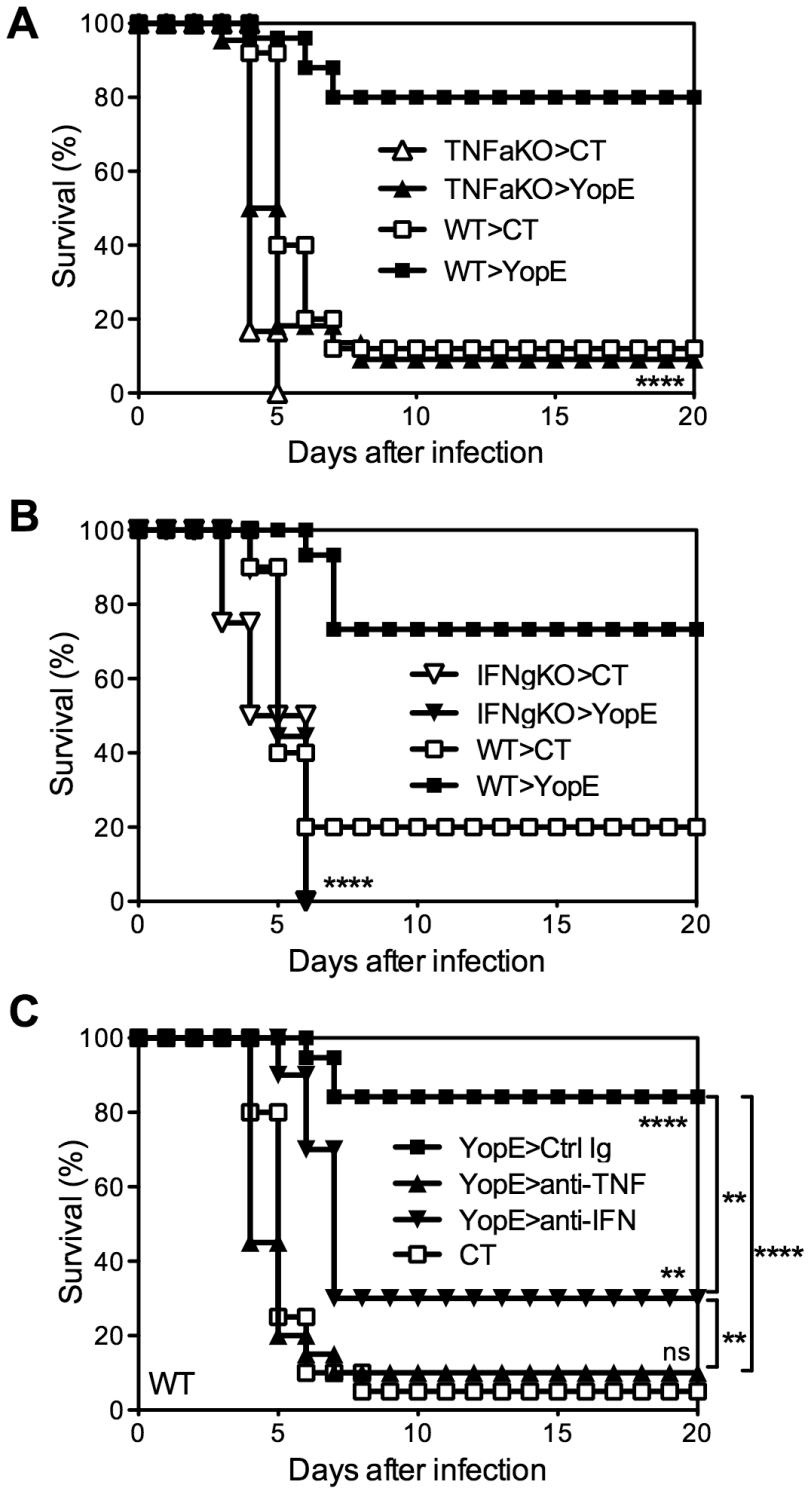
TNFα and IFNγ are critical for T cell-mediated protection against *Y. pestis*. (A and B) Wild-type (WT), TNFα-deficient (TNFaKO), IFNγ-deficient (IFNgKO) C57BL/6 mice were immunized intranasally with CT adjuvant alone or CT mixed with YopE_69–77_ peptide and then challenged intranasally with 20 MLD *Y. pestis* strain D27. In comparison with YopE_69–77_–immunized WT mice (n = 15–25), YopE_69–77_–immunized TNFα-deficient (n = 22) and IFNγ-deficient (n = 18) mice exhibited significantly reduced survival. Data were pooled from 3–5 independent experiments. (C) Wild-type C57BL/6 mice were immunized intranasally with CT alone or CT mixed with YopE_69–77_ peptide and then challenged intranasally with 20 MLD *Y. pestis* strain D27. One day before the challenge, the YopE_69–77_-immunized mice received neutralizing mAb specific for TNFα (anti-TNF), IFNγ (anti-IFN), or an isotype-matched mAb (Ctrl Ig). In comparison with CT-immunized mice (n = 20), YopE_69–77_-immunized mice treated with isotype-matched mAb (n = 19) or IFNγ-neutralizing mAbs (n = 10) but not TNFα-neutralizing mAb (n = 20) were protected against *Y. pestis* challenge. Data were pooled from 4 independent experiments.

To investigate roles for cytokines in CD8 T cell-mediated protection against *Y. pestis*, we immunized TNFα- or IFNγ-deficient mice with YopE_69–77_ and then challenged with 20 MLD of *Y. pestis* strain D27. While most YopE_69–77_-immunized wild-type mice survived the challenge, almost all YopE_69–77_-immunized TNFα-deficient mice and IFNγ-deficient mice succumbed (Figure 4A and 4B). Notably, the wild-type and IFNγ-deficient mice generated comparable frequencies of YopE_69–77_-specific CD8 T cells after immunization, and despite their impaired survival, the TNFα-deficient mice consistently generated higher frequencies of specific cells (data not shown), implying that cytokine deficiency does not quantitatively impair the expansion and/or persistence of YopE_69–77_-specific CD8 T cells. These results suggest that TNFα and IFNγ play critical roles during YopE_69–77_-specific CD8 T cell-mediated protection against pulmonary *Y. pestis* infection.

To assess whether protection mediated by YopE_69–77_-specific CD8 T cells requires cytokines at the time of challenge, YopE_69–77_-immunized wild-type mice were treated with neutralizing mAb specific for TNFα or IFNγ. Control mice were treated with an isotype-matched mAb of irrelevant specificity. As shown in Figure 4C, while mice treated with mAb to neutralize IFNγ at the time of challenge displayed decreased but still significant protection (p<0.01), nearly all mice treated with mAb to neutralize TNFα succumbed. These results suggest that production of TNFα is particularly critical at the time of pulmonary *Y. pestis* challenge.

### TNFα produced by either macrophages/neutrophils or T cells is dispensable for YopE_69–77_-specific CD8 T cell-mediated protection against *Y. pestis*

**Figure 5:**
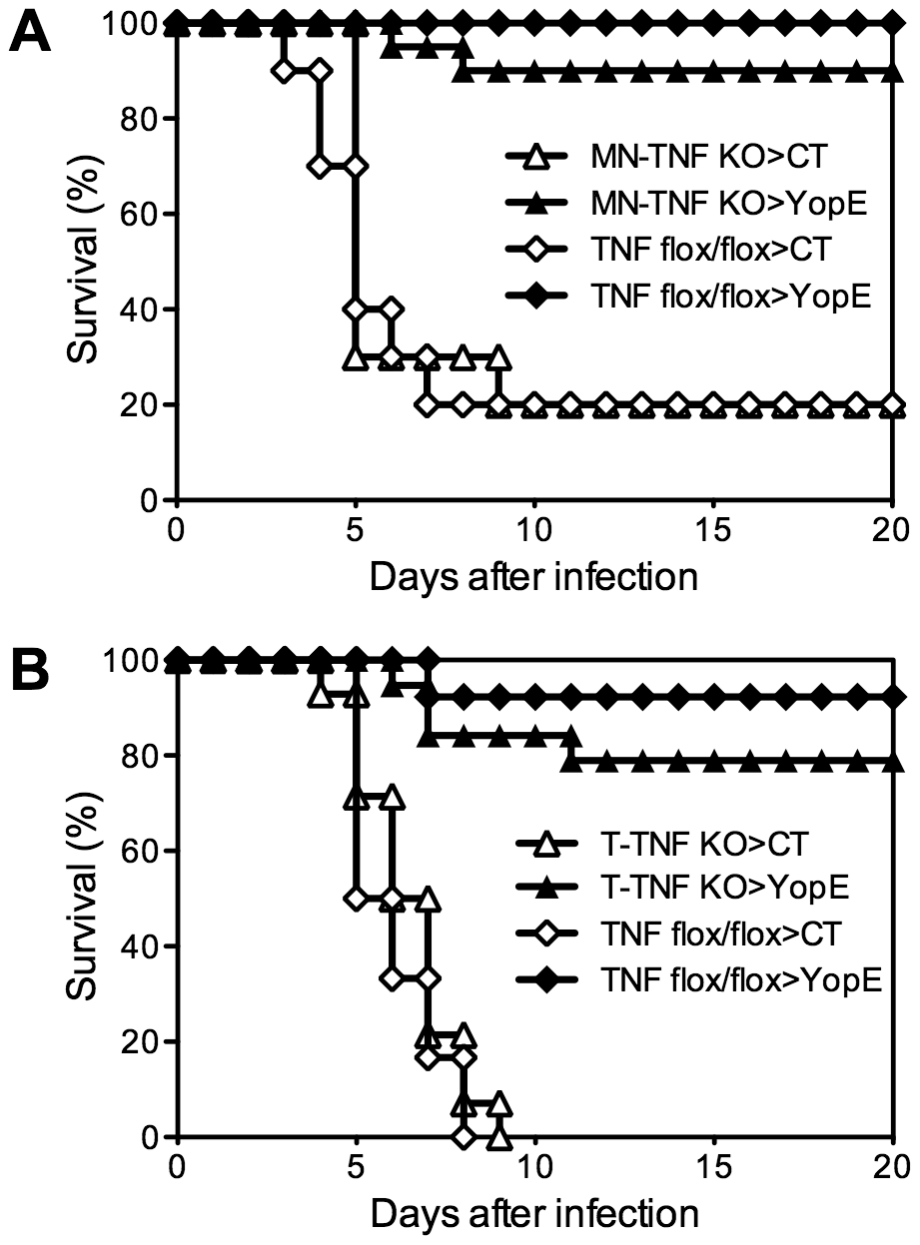
Selective depletion of TNFα from either macrophages/neutrophils or T cells does not impact the protection conferred by YopE_69–77_ immunization. Mice with (A) monocyte/neutrophil-specific (MN-TNF KO) or (B) T cell-specific (T-TNF KO) deletion of TNFα were immunized with CT adjuvant alone or CT mixed with YopE_69–77_ peptide and challenged intranasally with 20 MLD *Y. pestis* strain D27. Littermate TNF-floxed (TNF flox/flox) mice were used as controls. In comparison with CT-immunized mice (n = 10–14), YopE_69–77_-immunized MN-TNF KO mice (n = 20) and T-TNF KO mice (n = 19) were protected against *Y. pestis* challenge (p<0.0001) with no significant difference from YopE_69–77_-immunized TNF flox/flox mice (n = 12–13 mice/group). Data were pooled from 3 (A) and 5 (B) independent experiments.

A variety of cell types can produce TNFα during the immune response to infection. To assess whether a specific cell type is responsible for producing the critical TNFα during CD8 T cell-mediated defense against *Y. pestis*, we performed studies with previously described mice possessing cell-type-specific deficiencies in TNFα expression [18]. Control mice and mice deficient for TNFα expression in either the macrophage/neutrophil lineage (MN-TNF KO) or the T cell lineage (T-TNF KO) were immunized with YopE_69–77_. Immunized TNF-floxed littermate control mice largely survived challenge with 20 MLD of *Y. pestis* strain D27 (Figure 5). Unexpectedly, both the MN-TNF KO mice and the T-TNF KO mice were also protected by YopE_69–77_ immunization, with 90% and 80% surviving the lethal challenge, respectively (Figures 5A and 5B, respectively). These data suggest that TNFα derived from either macrophages/neutrophils or T cells can suffice to confer YopE_69–77_-mediated protection against pulmonary *Y. pestis* challenge.

### Neither TNFα nor IFNγ production by T cells is critical for YopE_69–77_-specific CD8 T cell-mediated protection against *Y. pestis*

**Figure 6:**
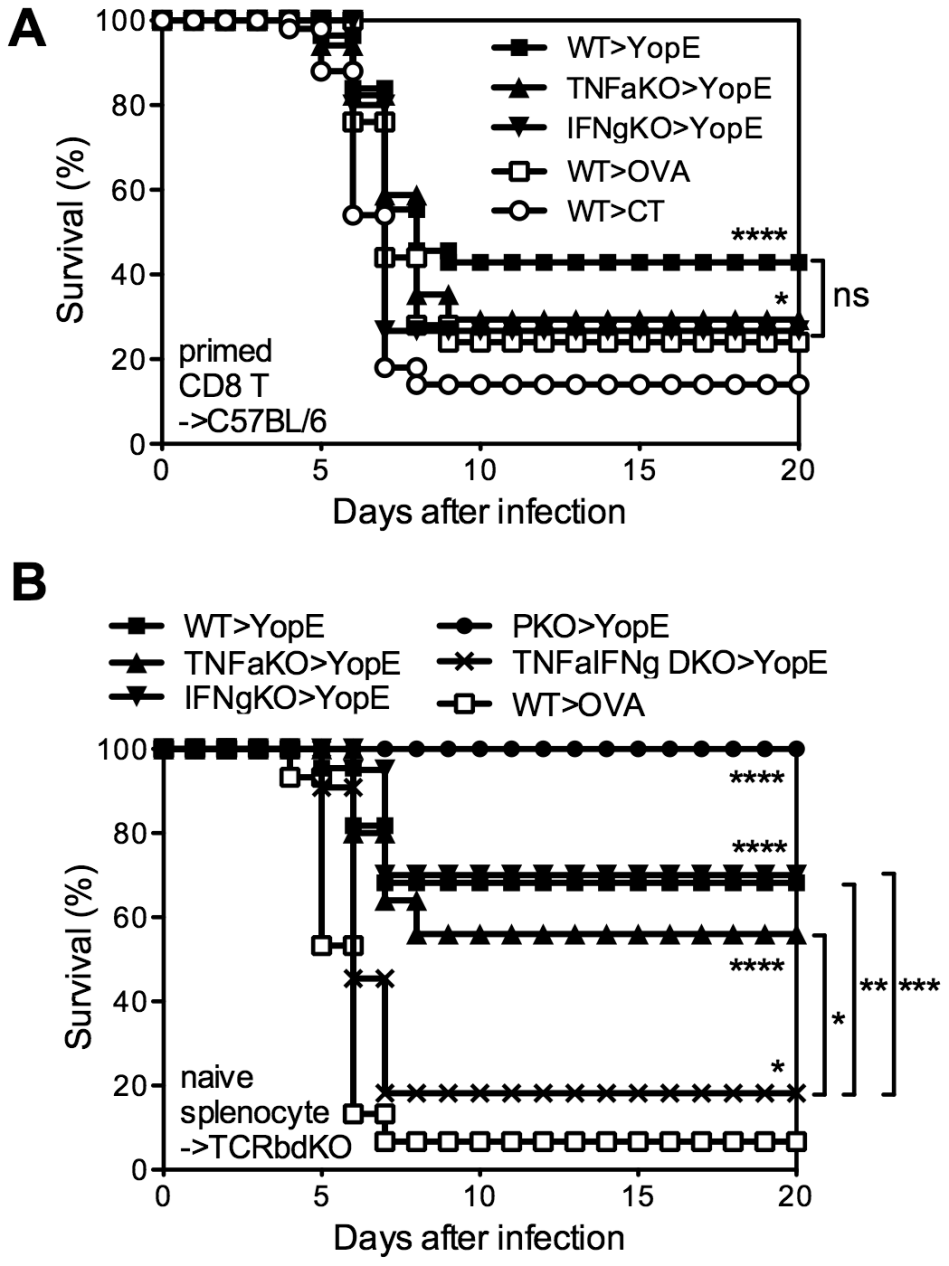
TNFα and IFNγ produced by YopE_69–77_-specific CD8 T cells have complementary roles during protection against *Y. pestis*. (A) Wild-type (WT), TNFα-deficient (TNFaKO) or IFNγ-deficient (IFNgKO) C57BL/6 mice were immunized with CT mixed with YopE_69–77_ peptide. WT mice immunized with CT adjuvant only or CT mixed with OVA_257–264_ peptide were used as controls. CD8+ splenocytes were then purified and transferred intravenously to naïve WT C57BL/6 mice, which were challenged intranasally with 20 MLD *Y. pestis* strain D27 the next day. In comparison with mice that received CD8+ T cells from CT-immunized WT mice (n = 50), mice that received CD8+ T cells from YopE_69–77_-immunized WT (n = 56), TNFα-deficient (n = 17) and IFNγ-deficient (n = 15) mice were protected against *Y. pestis* challenge. Notably, mice that received CD8+ T cells from OVA_257–264_-immunized WT mice (n = 25) were also protected (p<0.05). Data were pooled from 7 independent experiments. (B) Splenocytes isolated from naïve WT, TNFαKO, IFNγKO, perforin-deficient (PKO) or TNFα/IFNγ-deficient (TNFαIFNγ DKO) mice were transferred to TCRβδ-deficient mice. The mice were then immunized with CT mixed with YopE_69–77_. Control mice received naïve WT splenocytes and were immunized with CT mixed with OVA_257–264_ peptide. Mice were then challenged intranasally with 20 MLD *Y. pestis* strain D27. In comparison with control mice (n = 15), YopE_69–77_ immunized mice that received WT (n = 22), TNFαKO (n = 25), IFNγKO (n = 20), or PKO (n = 11) splenocytes were protected against *Y. pestis* challenge. YopE_69–77_-immunized mice that received TNFαIFNγ DKO splenocytes were also protected (n = 11) but the survival was significantly lower in comparison with the mice received WT, TNFαKO or IFNγKO splenocytes. Data were pooled from 3 independent experiments.

To definitively assess whether CD8 T cells are required to produce TNFα or IFNγ, we next assayed protection conferred by primed CD8 T cells isolated from YopE_69–77_-immunized TNFα- or IFNγ-deficient mice. Specifically, wild-type, TNFα-deficient, or IFNγ-deficient mice were immunized with YopE_69–77_ and their CD8+ splenic T cells were purified by magnetic beads and injected intravenously into naïve wild-type recipient mice, which were then challenged with 20 MLD of *Y. pestis* strain D27. The mice that received CD8+ T cells from YopE_69–77_-immunized wild-type mice showed significantly improved survival as compared with mice that received CD8+ T cells from control adjuvant-immunized mice (p<0.0001), with 43% of the mice surviving (Figure 6A). Mice that received CD8+ T cells from YopE_69–77_-immunized TNFα- or IFNγ-deficient mice displayed more modest but significant protection (both p<0.05), with 29% and 27% surviving the challenge, respectively. Thus, YopE_69–77_-specific CD8 T cells lacking the capacity to produce either TNFα or IFNγ can confer significant protection against *Y. pestis*.

In our experience, the transfer of primed T cells followed shortly thereafter by an infectious challenge can sometimes provide non-specific protection (data not shown), perhaps owing to the potential of effector and memory CD8 T cells to rapidly secrete IFNγ in response to IL-12 and IL-18 in the absence of cognate antigen [19]. In fact, the mice that received CD8+ T cells from OVA_257–264_-immunized wild-type mice displayed some protection, with 24% surviving the challenge (Figure 6A). Moreover, the protection in this primed T cell transfer model was relatively weak, as compared with our prior studies of intact mice, thus making it difficult to study the mechanism. Additionally, these YopE_69–77_-specific CD8 T cells were primed in an environment where cytokine was deficient. In an attempt to overcome these possible caveats to the experiments shown in Figure 6A, we next developed a model to study cytokine-deficient T cells that were primed *in situ*. First, we isolated naïve splenocytes from wild-type or gene-deficient mice and transferred them to TCRβδ-deficient mice, which lack T cells, thus creating chimeric mice in which primed T cells could only arise from cytokine-deficient donor splenocytes. Then, we immunized and challenged these splenocyte chimeras. The chimeras that received wild-type splenocytes showed significant YopE-specific protection (p<0.0001); 70% of the YopE_69–77_-immunized mice survived and only 7% of the control OVA_257–264_-immunized mice survived (Figure 6B). All the YopE_69–77_-immunized mice that received splenocytes from perforin-deficient mice also survived, further confirming that perforin is dispensable for protection against *Y. pestis* mediated by primed CD8 T cells (Figure 6B). YopE_69–77_-immunized mice that received splenocytes from TNFα-deficient or IFNγ-deficient mice also displayed significant protection (both p<0.0001), exhibiting 56% and 70% survival, respectively. Thus, mouse models employing either the transfer of primed CD8+ T cells (Figure 6A) or the priming of CD8+ T cells *in situ* (Figure 6B) demonstrate that YopE_69–77_-specific CD8 T cells lacking the capacity to produce either TNFα or IFNγ can confer significant protection against *Y. pestis*.

### TNFα and IFNγ produced by YopE_69–77_-specific CD8 T cells show complementary roles in protecting against *Y. pestis*

The observation that YopE_69–77_-specific CD8 T cells lacking either TNFα or IFNγ exhibit slightly impaired protection suggested that the TNFα and IFNγ produced by CD8 T cells may have complementary roles. To explore this possibility, we generated TNFα and IFNγ double-deficient (TNFα/IFNγ-deficient) mice. We observed that T cell-deficient mice that received splenocytes from TNFα/IFNγ-deficient mice were poorly protected by YopE_69–77_ immunization (Figure 6B), with only 18% surviving lethal *Y. pestis* challenge. The observation that further removing the remaining cytokine from either the TNFα- or IFNγ-deficient mice leads to a significant and substantial decrease in survival suggests complementary roles for TNFα and IFNγ produced by CD8 T cells.

**Figure 7:**
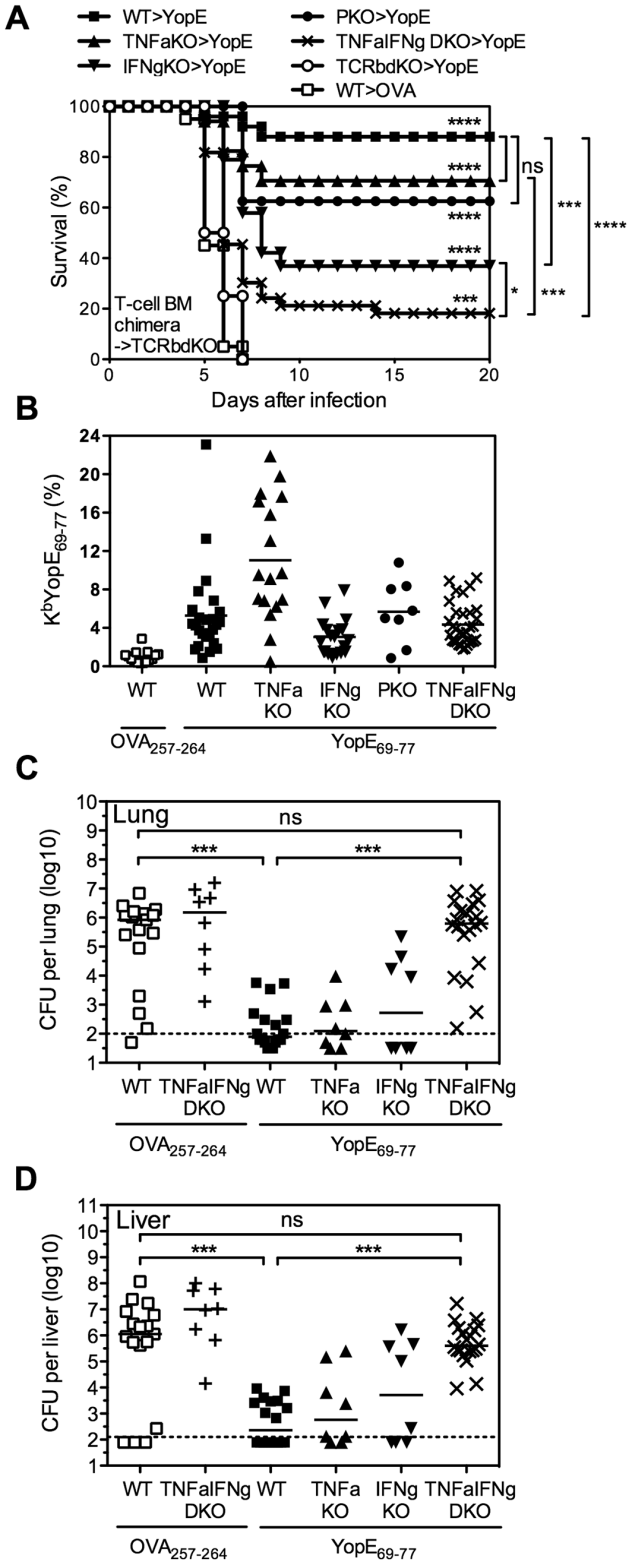
YopE_69–77_-specific CD8 T cells lacking the capacity to produce TNFα and IFNγ fail to protect mice and control bacterial burden. TCRβδ-deficient (TCRbdKO) mice were lethally irradiated and reconstituted with 75% TCRβδKO bone marrow cells and 25% of either WT, TNFαKO, IFNγKO, PKO, or TNFαIFNγ DKO bone marrow cells. Six weeks later they were immunized with CT mixed with YopE_69–77_ or OVA_257–264_ and then challenged intranasally with 20 MLD *Y. pestis* strain D27. (A) Survival. In comparison with OVA_257–264_-immunized mice reconstituted with WT T cells (n = 20), the YopE_69–77_-immunized chimeric mice reconstituted with WT (n = 25), TNFαKO (n = 17), IFNγKO (n = 19), PKO (n = 8) or TNFαIFNγ DKO (n = 33) T cells all showed significant protection. (B) The percentage of CD8+ T cells that stained positive for MHC class I tetramer K^b^YopE_69–77_ in PBL on the day before challenge. Solid bar depicts the mean. All groups of chimeric mice that were immunized with YopE_69–77_ had significantly increased frequency of K^b^YopE_69–77_+CD8+ T cells in compared with the chimeric mice immunized with OVA_257–264_ (p<0.001). YopE_69–77_-immunized chimeric mice reconstituted with TNFαKO T cells had significantly higher frequency of K^b^YopE_69–77_+CD8+ T cells in compared with YopE_69–77_-immunized chimeric mice reconstituted with WT T cells (p<0.01). Data for (A) and (B) are pooled from 6 independent experiments. (C and D) Bacterial burden in lung (C) and liver (D) tissues was measured at day 4 after challenge (Kruskal-Wallis test). Data are pooled from 3 independent experiments. Solid bar depicts median; broken line depicts the limit of detection.

In the splenocyte chimera model shown in Figure 6B, the cytokine-deficient splenic T cells had developed in the thymuses of cytokine-deficient mice, perhaps imprinting them with altered functions. To overcome this potential concern, a final set of studies was performed using mixed bone marrow chimeras. Lethally irradiated TCRβδ-deficient mice were reconstituted with a mixture of bone marrow cells isolated from gene-deficient or wild-type mice. In each case, 75% of the bone marrow cells came from TCRβδ-deficient mice, thereby enabling reconstitution of wild-type elements of all leukocyte populations except for T cells. The remaining 25% of the bone marrow cells came from wild-type mice or gene-deficient mice, thereby enabling the development of wild-type or gene-deficient T cells, respectively. Six weeks after reconstitution, the mice were immunized and then challenged with 20 MLD of *Y. pestis* strain D27. As shown in Figure 7A, 88% of the YopE_69–77_-immunized mice reconstituted with wild-type T cells were protected, while all of the OVA_257–264_-immunized mice reconstituted with wild-type T cells succumbed. Likewise, all YopE_69–77_-immunized mice reconstituted without T cells (i.e. mice that received only TCRβδ-deficient bone marrow) succumbed to challenge infection. Consistent with Figures 6, the YopE_69–77_-immunized chimeric mice reconstituted with TNFα-, IFNγ- or perforin-deficient T cells all showed significantly improved protection when compared with mice lacking T cells (all p<0.0001), indicating that production of TNFα-, IFNγ- or perforin by T cells is not absolutely essential. Indeed, the degree of protection observed in YopE_69–77_-immunized chimeric mice reconstituted with TNFα- or perforin-deficient T cells did not differ significantly from that observed in mice reconstituted with wild-type T cells (Figure 7A). Although the YopE_69–77_-immunized chimeric mice reconstituted with IFNγ-deficient T cells displayed significantly increased protection in comparison with mice lacking T cells, the degree of protection was significantly reduced in comparison with mice reconstituted with wild-type T cells (p<0.001, Figure 7A). Notably, survival was further reduced in mice reconstituted with TNFα/IFNγ-deficient T cells (p<0.05 in compared with mice reconstituted with IFNγ-deficient T cells), with only 18% of mice surviving the lethal challenge (Figure 7A).

It is worth noting that we confirmed the efficiency of bone marrow reconstitution and YopE immunization in mice prior to *Y. pestis* infection by measuring the frequency of CD4 T cells, CD8 T cells, and YopE_69–77_-specific CD8 T cells in the peripheral blood. In spite of slightly lower frequency of CD8 T cells in the peripheral blood of mice reconstituted with TNFα-, IFNγ- or TNFα/IFNγ-deficient T cells than that of mice reconstituted with wild-type T cells (data not shown), the frequency of YopE_69–77_-specific CD8 T cells, as measured by K^b^YopE_69–77_ tetramer, was not lower than that of mice reconstituted with wild-type T cells (Figure 7B). In fact, mice reconstituted with TNFα-deficient T cells generated even more YopE_69–77_-specific CD8 T cells than mice reconstituted with wild-type T cells, even though they exhibited reduced survival (Figure 7A).

Subsequently, we generated bone marrow chimeras, immunized and challenged them as described above, and then euthanized mice at day 4 after challenge to measure bacterial burden. This analysis revealed significantly decreased bacterial CFU in lung (Figure 7C) and liver (Figure 7D) tissues of YopE_69–77_-immunized chimeric mice reconstituted with wild-type, TNFα- or IFNγ-deficient T cells, as compared with OVA_257–264_-immunized chimeric mice reconstituted with wild-type T cells. Thus, deficient production of either TNFα or IFNγ by T cells does not impair their ability to control bacteria. In contrast, we observed significantly increased numbers of bacterial CFU in lung and liver tissues of YopE_69–77_-immunized chimeric mice reconstituted with TNFα/IFNγ-deficient T cells, as compared with YopE_69–77_-immunized chimeric mice reconstituted with wild-type T cells (Figure 7C and 7D). In fact, the burden of YopE_69–77_-immunized chimeric mice reconstituted with TNFα/IFNγ-deficient T cells achieved levels approaching those of OVA_257–264_-immunized chimeric mice reconstituted with either wild-type or TNFα/IFNγ-deficient T cells, which were not protected (Figure 7A).

## Discussion

In this study, we conducted experiments to better understand the mechanisms of immunization-induced protective CD8 T cell responses and their relative contributions to defense against pulmonary *Y. pestis* infection. We extended our prior work [13] and demonstrated that immunization with YopE_69–77_ suffices to confer remarkable protection against pigmentation locus-deficient *Y. pestis* strain D27 (Figures 1A and 1B). YopE_69–77_-immunized mice can withstand as high as 200 MLD challenge (Figure 1B). Intranasal administration of *Y. pestis* strain D27 causes mortality in mice but does not cause the fulminant pneumonia characteristic of fully virulent *Y. pestis* [20]. However, we also found that YopE_69–77_-immunized mice were significantly protected against intranasal infection with the fully virulent *Y. pestis* strain CO92 (Figure 1C). To our knowledge, this is the first report that T cell-mediated immunity can protect against fully virulent *Y. pestis* infection. Notably, the protection was incomplete, suggesting that YopE_69–77_-specific CD8 T cells alone may be insufficient to combat fully virulent *Y. pestis* strains.

Taking advantage of the robust protection observed in mice infected with 20 MLD *Y. pestis* strain D27, we manipulated immune responses to identify effector functions that are critical for CD8 T cell-mediated anti-*Yersinia* immunity *in vivo*. Researchers typically use gene-deficient mice, adoptive cell transfer models and Ab-mediated depletion methods to demonstrate roles for different effector functions of cellular immunity during host defense against microbial infections. There are caveats to each approach and determining the relative contribution of each specific effector function by any particular type of immune cell can be technically challenging. First, effector functions usually are not unique to a particular cell type. Both CD8 T cells and NK cells utilize perforin-mediated cell killing, whereas TNFα and/or IFNγ can be produced by CD4 T cells, CD8 T cells, NK cells, and some innate cells such as macrophages and neutrophils. Second, the removal of any particular effector function can inadvertently alter cell differentiation, homeostasis, and/or the immunodominance hierarchies of Ag-specific cells. For example, IFNγ-deficient and perforin-deficient mice exhibit altered expansion, contraction and immunodominance of Ag-specific CD8 T cells during *Listeria* infection [21]. Third, effector and memory Ag-specific CD8 T cells have the potential to rapidly secrete IFNγ in response to IL-12 and IL-18 in the absence of cognate Ag [19], hence transferring primed effector/memory Ag-specific cells to mice followed by infection could confer innate protection in an Ag nonspecific manner. In an effort to address these complexities and overcome the limitations of any single methodology, we used multiple approaches to examine the contributions of potential effector mechanisms used by CD8 T cells to combat septic pneumonic plague.

YopE_69–77_-immunized perforin-deficient mice, which display severely impaired Ag-specific cytotoxic activity *in vivo* (Figure 2), are resistant to lethal *Y. pestis* and *Y. pseudotuberculosis* challenge (Figure 3). Likewise, the levels of protection provided by wild-type and perforin-deficient YopE_69–77_-specific CD8 T cells are indistinguishable in T cell chimeric mice (Figure 6B and 7A). These data strongly suggest that perforin does not contribute substantially to CD8 T cell-dependent adaptive host defense against *Yersinia*. In contrast, a recent publication by Bergman et al. suggested that perforin can be critical for protection against *Y. pseudotuberculosis* infection [15]. They studied naïve mice infected with an attenuated strain of *Y. pseudotuberculosis* and reported that perforin deficiency impaired protection, as evidenced by greater bacterial burden in the spleens and livers. When the surviving perforin-deficient mice were challenged with a virulent strain of *Y. pseudotuberculosis*, the “immunized” perforin-deficient mice only exhibited significantly impaired protection if animals without detectable bacteria were excluded from the analysis. Since we found no evidence that perforin deficiency impairs either bacterial clearance or survival in YopE_69–77_-immunized mice, the combined data suggest that perforin may be important for primary defense against *Yersinia* but can be dispensable for adaptive defense.

While perforin deficiency has little impact on protection mediated by YopE_69–77_-specific CD8 T cells, we note that perforin deficiency did not completely abolish all cytotoxicity in our model (Figure 2B). Thus, we cannot exclude the possibility that the residual cytotoxicity, which is independent of perforin, may contribute to adaptive defense against *Yersinia*. Future studies will need to assess whether such other cytotoxic pathways, for example, Fas/FasL and TNF/TNF receptors, contribute to YopE_69–77_-specific CD8 T cell-mediated protection.

In contrast to perforin deficiency, cytokine deficiency dramatically impairs anti-*Yersinia* immunity mediated by YopE_69–77_-specific CD8 T cells. Data presented in this study highlights the complexity of cytokine-mediated protection. YopE_69–77_-immunized mice that completely lack the ability to produce either TNFα or IFNγ succumb to infection, suggesting that each of these cytokines are absolutely required for CD8 T cell-mediated anti-*Yersinia* immunity (Figures 4A and 4B). However, the data in Figure 4C reveal that IFNγ is not absolutely required for protection at the time of challenge. It implies that IFNγ may have a role in the development of YopE_69–77_-specific CD8 T cells that have the capacity to combat *Y. pestis*. Moreover, the data in Figure 6 reveal that IFNγ production by T cells is not required for resistance to *Y. pestis*, implying that cell types other than T cells must be capable of producing enough IFNγ to confer modest yet significant protection. In contrast, chimeric mice in which the T cells cannot produce IFNγ exhibit significant impairments in survival after YopE immunization and *Y. pestis* challenge (Figure 7A). While we cannot completely exclude the possibility that cell reconstitution in mice deficient for T cell-derived IFNγ is suboptimal, the frequency of YopE_69–77_-specific CD8 T cells in the peripheral blood prior to challenge is similar in wild-type mice and chimeric mice whose T cells cannot produce IFNγ (Figure 7B). Since other cells in the environment are capable of producing IFNγ in that model, the data suggest that a cell-intrinsic defect resulting from the loss of IFNγ influences some quality of differentiating CD8 T cells. One possibility is that mice whose T cells cannot produce IFNγ may have reduced TNFα production or responsiveness. Indeed, IFNγ has been shown to enhance TNF receptor expression [22].

In contrast to IFNγ, Ab-mediated depletion of TNFα at the time of *Y. pestis* challenge abrogates nearly all protection in YopE_69–77_-immunized mice, suggesting that TNFα is absolutely required for protection at the time of challenge (Figure 4C). Cells of the macrophage, neutrophil and T cell lineages can each produce TNFα. However, TNFα production by T cells is not absolutely critical for resistance to *Y. pestis*, implying that cell types other than CD8 T cells must be capable of producing enough TNFα to confer modest yet significant protection (Figures 5B, 6 and 7). We found that TNFα from macrophages/neutrophils is not absolutely required for protection either, at least not when the mice have intact IFNγ production (Figure 5A). These observations imply that TNFα from one cell type can substitute for the loss of TNFα in another cell type in pulmonary *Y. pestis* infection. Alternatively, residual production of TNFα from cells other than T cells and macrophages/neutrophils could also contribute to protection.

In contrast to the dramatic impact of Ab-mediated depletion of TNFα at the time of challenge, the impact of depleting CD8 T cell-derived TNFα seems to be modest (Figures 6 and 7). One caveat is that we repeatedly observed a higher frequency of YopE_69–77_-specific CD8 T cells in the peripheral blood of mice whose T cells could not produce TNFα (Figure 7B and data not shown). Thus, it is conceivable that impairments in protection caused by the absence of T cell-derived TNFα can be restored, at least partially, by an increase in number of YopE_69–77_-specific CD8 T cells.

While depleting either TNFα or IFNγ from CD8 T cells impairs protection to a certain extent, simultaneously depleting both cytokines additionally compromises protection. Specifically, chimeric mice whose T cells can produce neither cytokine display significantly reduced survival when compared with mice whose T cells cannot produce either TNFα or IFNγ (Figures 6B and 7A). Some TNFα production from either T cells or non-T cells, in concert with intact IFNγ production, appears to be sufficient for near optimal protection (Figure 5). However, the data suggest that TNFα and IFNγ production by CD8 T cells work complementarily to confer optimal protection. One likely mechanism by which these cytokines contribute to host survival is through the control of bacterial burden (Figures 7C and 7D). TNFα and IFNγ are known to activate macrophages and neutrophils to produce reactive nitrogen and oxygen intermediates that assist in the control of intracellular bacteria. Adding purified TNFα and IFNγ to the macrophage cultures can overcome *Y. pestis*-induced suppression of macrophage anti-bacterial activity *in vitro* [23]. These cytokines can also stimulate neutrophils to secret antimicrobial molecules to control extracellular bacteria.

Although depleting both TNFα and IFNγ produced by T cells dramatically compromises the protection mediated by YopE_69–77_-specific CD8 T cells, it does not completely abolish the protection. We did observe a minimal of 20% host survival, which is significant in comparison with OVA_257–264_-immunized wild-type mice and reproducible. This finding implies that while CD8 T cell-derived TNFα and IFNγ are critical for host defense against *Y. pesitis*, they may not be the sole players. Despite the observation that perforin is dispensable for CD8 T cell-mediated protection against *Y. pestis* (Figure 3), its function may contribute to host survival when the two key effector functions of CD8 T cells are absent. Moreover, other mediators that CD8 T cells can produce during infection may also contribute to protection. For example, CD8 T cell production of macrophage inflammatory protein-1α (MIP-1α/CCL3) is required for anti-*Listeria* immunity [24], [25].

One could argue that in our splenocyte transfer and bone marrow chimera models (Figures 6B and 7A, respectively), CD4 T cells share the same defect with CD8 T cells. However, we found that CD4 T cells are not required for YopE_69–77_-mediated protection, since YopE_69–77_-immunized CD4-deficient mice were protected as well as YopE_69–77_-immunized wild-type mice (unpublished data). Thus, we think it unlikely that a deficiency of CD4 T cell-derived cytokines accounts for the lost of protection we observe in these models.

CD8 T cells can utilize a variety of mechanisms to protect against infectious agents. Understanding the effective and critical functions of the CD8 T cell response against pathogens that cause septic bacterial pneumonias and defining the combinations of effector functions that optimally combat such pathogens should improve our ability to design effective vaccines. Our study shows that YopE_69–77_-specific CD8 T cells exhibit perforin-dependent cytotoxic activity *in vivo*, but this effector function does not appear to be required for anti-*Yersinia* immunity. In contrast, production of TNFα and IFNγ is essential for CD8 T cell-mediated protection. Interestingly, the time at which these cytokines must be produced appears to differ, as does their essential cellular sources. Overall, the data suggest that CD8 T cell-derived TNFα and IFNγ exert complementary functions and CD8 T cells require both cytokines to provide optimal defense against pulmonary *Y. pestis* infection. Our study further suggests cytokine-producing CD8 T cells may be a valuable addition to subunit plague vaccines and assays detecting Ag-specific TNFα production may be a particularly useful correlate of plague vaccine efficacy. Given that septic bacterial pneumonias are a leading cause of death, it will be interesting to determine whether cytokine production, rather than cytolysis, is generally more important for T cell-mediated defense against septic bacterial pneumonias.

## Materials and Methods

### Ethics statement

All animal studies were conducted in accordance with the Guide for Care and Use of Laboratory Animals of the National Institutes of Heath and approved by Trudeau Institute Animal Care and Use Committee (IACUC protocols # 02-022 and 02-161).

### Mice

Wild-type C57BL/6, B6.129S7-*Ifng^tm1Ts^* (IFNγ-deficient), B6.129S-*Tnf^tm1Gkl^* (TNFα-deficient), C57BL/6-*Prf1^tm1Sdz^* (perforin-deficient), B6.129P2-*Tcr^btm1Mom^ Tcrd^tm1Mom^* (TCRβδ-deficient), B6.SJL-*Ptprc^a^ Pepc^b^*/BoyJ (CD45.1) mice were purchased from The Jackson Laboratory (Bar Harbor, ME) and then bred in the specific-pathogen-free Trudeau Institute Animal Breeding Facility. TNFα/IFNγ double-deficient mice were generated at Trudeau Institute. Mice deficient for TNFα production in macrophage and neutrophils (MN-TNF KO mice) or T cells (T-TNF KO mice) were generated by crossing mice with TNF “floxed” genes to mice carrying the LysM-Cre or CD4-Cre transgenes, respectively [18]. All strains are on the C57BL/6 background. Experimental mice were matched for age and sex, and first immunized between 6 and 10 weeks of age.

### Bacteria and infections

Pigmentation locus-deficient *Y. pestis* strain KIM D27 was originally provided by Dr. Robert Brubaker (Michigan State University, East Lansing, MI). Strain D27 bacilli from frozen glycerol stocks were grown overnight at 26°C in Bacto heart infusion broth (Difco Laboratories, Detroit, MI) supplemented with 2.5 mM CaCl_2_. Cultures were then diluted to an OD of 0.1 at 620 nm, regrown for 3–4 h at 26°C, quantified by OD measurement, and then washed and resuspended in saline at the desired concentration. The numbers of bacterial CFU in the inocula were confirmed by plating. Infections were performed by applying 30 µl to the nares of mice that were lightly anesthetized with isoflurane. The intranasal median lethal dose (MLD) for strain D27 is approximately 1×10^4^ CFU when the bacteria are grown and administered as described above [26].

The fully virulent pigmentation locus-positive *Y. pestis* strain CO92 NR-641 was obtained through the NIH Biodefense and Emerging Infections Research Resources Repository, NIAID, NIH. Bacteria from frozen glycerol stocks were grown overnight at 26°C in Bacto heart infusion broth. Cultures were then diluted to an OD of approximately 0.1 at 620 nm, regrown for 2.5–3 h at 26°C, quantified by OD measurement, and prepared at the desired concentration. The numbers of bacterial CFU in the inocula were confirmed by plating. Infections were performed by applying 25 µl to the nares of mice that were lightly anesthetized with isoflurane. Preliminary studies established that the intranasal MLD for strain CO92 is approximately 1×10^3^ CFU when the bacteria are grown and administered as described above.

*Y. pseudotuberculosis* serotype O:1 strain 32777 bacilli from frozen glycerol stocks were grown overnight at 26°C in Luria-Bertani medium to reach an OD of approximately 1.2 at 600 nm. The cultures were then washed and resuspended in saline to achieve the desired concentration. For intragastric infections, mice were fasted for 20–21 h and then inoculated with 200 µl of *Y. pseudotuberculosis* through a 20-gauge feeding needle. For intravenous infections, mice were injected with 200 µl of *Y. pseudotuberculosis* via the tail vein. The intragastric and intravenous MLD for strain 32777 is approximately 5×10^8^ CFU and 12 CFU, respectively, when the bacteria are grown and administered as described above.

### Immunization with YopE and ovalbumin peptides

Unless otherwise indicated, mice were lightly anesthetized with isoflurane and immunized intranasally on days 0, 7 and 21 with 15 µl saline solution containing 1 µg cholera toxin (CT; List Biological Laboratory, Campbell, CA) as adjuvant and 1 µg YopE_69–77_ peptide (H_2_N-SVIGFIQRM-OH; New England Peptide, Gardner, MA) or control OVA_257–264_ peptide (H_2_N-SIINFEKL-OH). Mice were then challenged with 20 or 200 MLD of *Y. pestis* strain D27, 10 MLD of *Y. pestis* strain CO92 or 10 MLD of *Y. pseudotuberculosis* strain 32777 at day 37. In some experiments, PBL were collected by mandibular bleeding at day 35 to assay Ag-specific CD8 T cells by flow cytometry.

### *In vivo* cytotoxicity assay

Splenocytes harvested from naïve CD45.1+ congenic mice were used as target cells. After lysing red blood cells using ammonium chloride buffer, the splenocytes were pulsed with either YopE_69–77_ or OVA_257–264_ peptide (10 µM) for 1 h at 37°C in complete medium (DMEM supplemented with 10% heat-inactivated FCS, 2 mM L-glutamine, 1 mM sodium pyruvate, 0.1 mM nonessential amino acids, 1% penicillin-streptomycin and 55 µM 2-mercaptoethanol). After washing and resuspending in Hank's balanced salt solution, YopE_69–77_-pulsed cells were incubated with 10 µM CFSE and OVA_257–264_-pulsed cells were incubated with 1 µM CFSE for 10z min in a 37°C water bath. CFSE labeling was then stopped by adding FBS to a final concentration of 20%. Cells were washed twice with complete medium and mixed at a 1∶1 ratio. A total of 2×10^7^ cells were then injected intravenously into immunized recipient mice. Spleen cells were collected 20–22 h later and stained with PE-conjugated anti-CD45.1 mAb, biotin-conjugated anti-CD45.2 mAb and allophycocyanin-conjugated streptavidin. The frequency of each CFSE-labeled population in the target cell gate (CD45.1+CD45.2−) was determined by flow cytometry. Cells injected into naïve C57BL/6 mice were used as controls. The percent specific lysis was determined by the following formula: [1-(ratio of cells recovered from naïve mice/ratio of cells recovered from infected mice)]×100. For YopE-specific lysis, the ratio of recovery of OVA_257–264_-pulsed cells to YopE_69–77_-pulsed cells = (percentage of CFSE^low^ cells)/(percentage of CFSE^high^ cells). For OVA-specific lysis, the ratio of recovery of YopE_69–77_-pulsed cells to OVA_257–264_-pulsed cells = (percentage of CFSE^high^ cells)/(percentage of CFSE^low^ cells).

### MHC class I tetramer reagent and staining

The allophycocyanin-conjugated MHC class I peptide tetramers K^b^YopE_69–77_ and K^b^ OVA_257–264_ were supplied by the NIH Tetramer Facility and the Trudeau Institute Molecular Biology Core Facility, respectively. PBL were obtained from mandibular blood collected in heparin-containing eppendorf tubes. Red blood cells were lysed by treatment with ammonium chloride. Enumeration of YopE_69–77_-specific CD8 T cells by K^b^YopE_69–77_ tetramer staining was described previously [13]. In brief, cells were treated with 1 µg Fc block (clone 2.4G2) for 10 min at 4°C, washed, and incubated with tetramers for 1 h at room temperature. After washing again, cells were stained with anti-CD4-FITC (clone RM4–5) and anti-CD8-PE (clone 53-6.7) for 30 min at 4°C. Data were collected on a BD Bioscience FACSCanto II and analyzed using FlowJo software.

### Measurement of survival and bacteria burden

For survival studies, mice were monitored at least once daily after infection. Unresponsive or recumbent mice were considered moribund and euthanized. For measurement of bacterial burden, mice were euthanized by carbon dioxide asphyxiation at the indicated day after infection. Liver and lung tissues were harvested and homogenized in saline. Serial dilutions of the homogenates were plated on blood agar and incubated at 26°C for 48 h.

### Cytokine neutralization

Mice were treated with 1 mg mAb specific for TNFα (clone XT3.11, rat IgG1) or IFNγ (clone XMG1.2, rat IgG1) diluted in PBS. The mAb were administered intraperitoneally on the day before challenge infection. Control mice received 1–1.6 mg of isotype-matched rat IgG1 mAb (clone HRPN).

### Cell purifications and transfers

For primed CD8^+^ T cell transfers, donor mice were immunized intranasally as described above on days 0, 7 and 21. At day 36, splenocytes were harvested, red blood cells were lysed and cell suspensions were applied to nylon wool columns to enrich T cells. After incubation at 37°C for 90 min, the nonadherent cells were eluted and CD8+ T cells were purified by magnetic activated cell sorting (MACS; Miltenyi Biotec Inc., Auburn, CA) according to the manufacturer's instructions. The purified cells were incubated at 37°C for 90 min followed by centrifugation to release beads from cells. The purity of CD8+ donor T cells was ∼95%. A total of 1×10^7^ CD8^+^ T cells were injected intravenously into naïve wild-type C57BL/6 recipient mice. The following day, the recipient mice were challenged with *Y. pestis* strain D27.

For naïve splenocyte transfers, donor splenocytes were isolated and red blood cells were lysed. After washing, 5×10^7^ cells were injected intravenously into naïve TCRβδ-deficient recipient mice. The recipient mice were then immunized and challenged with *Y. pestis* strain D27 as described above.

### Mixed bone marrow chimeras

TCRβδ-deficient mice were lethally irradiated (950 rad provided in two doses) and reconstituted with a total of 1×10^7^ donor bone marrow cells. The donor cells comprised bone marrow cells from TCRβδ-deficient mice mixed with bone marrow cells from wild-type C57BL/6 mice or the indicated gene-deficient mice at a 3 to 1 ratio. Chimeric mice were allowed to reconstitute for 6 weeks before they were immunized and challenged with *Y. pestis* strain D27.

### Statistics

Statistical analyses were performed using the computer program Prism 5 (GraphPad Software). Survival data were analyzed by log-rank tests. Bacterial burden data were analyzed by nonparametric Kruskal-Wallis test followed by Dunn's post test; CFU that fell below the detection limit of our assay were assigned values 0.2 log below the detection limit. All other data were analyzed by Student *t* test or one-way ANOVA as indicated. *p<0.5, **p<0.01, ***p<0.001, ****p<0.0001; ns, not significant.
